# Medium-Term Outcomes in COVID-19

**DOI:** 10.3390/jcm11072033

**Published:** 2022-04-05

**Authors:** Zaki Akhtar, Sumeet Sharma, Ahmed I. Elbatran, Lisa W. M. Leung, Christos Kontogiannis, Michael Spartalis, Alice Roberts, Abhay Bajpai, Zia Zuberi, Mark M. Gallagher

**Affiliations:** 1 Department of Cardiology, Ashford and St Peter’s Hospitals NHS Trust, Surrey KT16 0PZ, UK; sumeetsharma@nhs.net (S.S.); alice.roberts@stgeorges.nhs.uk (A.R.); abhay.bajpai@nhs.net (A.B.); mark_m_gallagher@hotmail.com (M.M.G.); 2 Department of Cardiology, St George’s University Hospital, London SW17 0QT, UK; a.i.elbatran@gmail.com (A.I.E.); lleung@sgul.ac.uk (L.W.M.L.); christos.kontogiannis@stgeorges.nhs.uk (C.K.); zia.zuberi@nhs.net (Z.Z.); 3 Department of Cardiology, Ain Shams University, Abbasiya Square, Cairo 11566, Egypt; 4 Department of Cardiology, National and Kapodistrian University of Athens, 10679 Athens, Greece; mspartalis@icloud.com

**Keywords:** COVID-19, QTc, mortality, R-R interval

## Abstract

COVID-19 causes severe illness that results in morbidity and mortality. Electrocardiographic features, including QT prolongation, have been associated with poor acute outcomes; data on the medium-term outcomes remain scarce. This study evaluated the 1-year outcomes of patients who survived the acute COVID-19 infection. Methods and Materials: Data of the 159 patients who survived the COVID-19 illness during the first wave (1 March 2020–18 May 2020) were collected. Patient demographics, laboratory findings and electrocardiography data were evaluated. Patients who subsequently died within 1-year of the index illness were compared to those who remained well. Results: Of the 159 patients who had survived the index illness, 28 (17.6%) subsequently perished within 1-year. In comparison to the patients that were alive after 1-year, the deceased were older (68 vs. 83 years, *p* < 0.01) and equally male (60.4% vs. 53.6%, *p* = 0.68), with a similar proportion of hypertension (59.5% vs. 57.1%, *p* = 0.68), diabetes (25.2% vs. 39.2%, *p* = 0.096) and ischaemic heart disease (11.5% vs. 7.1%, *p* = 0.54). The QTc interval for the alive and deceased patients shortened by a similar degree from the illness to post-COVID (−26 ± 33.5 vs. −20.6 ± 30.04 milliseconds, *p* = 0.5); the post-COVID R-R interval was longer in the alive patients compared to the deceased (818.9 ± 169.3 vs. 761.1 ± 61.2 ms, *p* = 0.02). A multivariate Cox regression analysis revealed that age (HR1.098 [1.045–1.153], *p* < 0.01), diabetes (HR3.972 [1.47–10.8], *p* < 0.01) and the post-COVID R-R interval (HR0.993 [0.989–0.996], *p* < 0.01) were associated with 1-year mortality. Conclusions: The COVID-19-associated mortality risk extends to the post-COVID period. The QTc does recover following the acute illness and is not associated with outcomes; the R-R interval is a predictor of 1-year mortality.

## 1. Introduction

The coronavirus disease-19 (COVID-19) is a multi-system illness caused by the severe acute respiratory syndrome coronavirus-2 infection (SARS-CoV-2). The acute effects of the infection on the cardiovascular system are well documented: acute cardiac injury has been reported [1], and arrhythmias are common [2,3]. Patients have also been found to have abnormal electrocardiographic features, including T-wave inversions [4], left bundle branch block [5] and QT prolongation [6,7]. These are associated with poor outcomes in the acute illness, particularly QT prolongation [6,7].

In the medium-term, ‘long-COVID’ has been described, which is characterised by chronic fatigue, shortness of breath (SOB), anxiety, depression, memory impairment and a loss of concentration [8,9]. The medium-term effects on the cardiovascular system are not as well documented. Evidence is emerging and demonstrative of a wide array of cardiovascular risk in the 1-year follow-up period. Postural orthostatic hypotension syndrome (POTS) and inappropriate sinus tachycardia have been reported in a proportion of patients with post-COVID syndrome [10], whilst heart failure, thromboembolic disease and cerebrovascular disorders are also concerns [11].

Electrocardiographically, there is minimal assessment on the post-COVID changes in patients who survived COVID-19. This is despite the compelling changes witnessed during the acute illness. It remains uncertain whether these changes resolve or whether they have any lasting implications. In the general population, QT prolongation is associated with long-term all-cause mortality [12], and although the association of this interval with mortality in the acute illness is well established, the association with medium-term outcomes is yet to be determined.

In this study, we evaluated the medium-term (1-year) outcomes of previously hospitalised COVID-19 patients, which were associated with QT-prolongation.

## 2. Method and Materials

This is a follow-up study of 160 hospitalised patients previously reported to have survived the COVID-19 illness during the first COVID-19 wave in the UK between 1 March–18 May 2020 [7]. All patients with available follow-up data at our institute (St Peter’s Hospital NHS trust, Chertsey, UK) were recruited, and patients whose follow-up data were not available were excluded. Of the 160 patients, 159 were included in this study; 1 patient was lost to the follow-up and therefore excluded. For these participants, data were collected retrospectively using the electronic patient records of demographics, electrocardiogram (ECG) information (pre-admission, on admission and within 1 year of discharge), 1-year outcome from the index episode (COVID-19 discharge) and biochemical and haematological profiles. Patient data from post-COVID clinics were also collated to establish the diagnosis of long-COVID. A comparison was performed of patients who died within the 1-year follow up period and those who remained alive. The study proposal was approved by the research ethics committee and complies with the principles of the Declaration of Helsinki.

### 2.1. ECG Analysis

On admission for COVID-19, all patients underwent a standard 12-lead ECG at 25 mm/s paper speed and amplitude of 10 mm/mV (MAC 5500 HD ECG system, GE healthcare, Chicago, IL, USA). These were stored in the patient electronic medical records on the hospital server. For all patients included in this study, this baseline ECG was retrieved from the electronic records and analysed. Of note, at the time of recording of the baseline ECG, the diagnosis of COVID-19 had not been established in any of the patients, so no specific therapy had commenced; the baseline ECG was not impacted by any COVID-19 pharmacological therapy.

The methodology of ECG analysis has been reported previously [7]. Briefly, basic information, including rhythm, rate, QRS duration and morphology and corrected QT interval (QTc), were derived from the baseline ECG. The QTc was calculated manually by a cardiologist: in each individual patient ECG, the longest QT interval (measured from the onset of the Q-wave to the T-wave offset [the point of intersection of the downward slope tangent-line with the baseline]) was identified [13]; correction was performed using Bazett’s formula [7]. Corrected QT clinical limits of >460 milliseconds (ms) for females and >450 ms for males were accepted as prolonged [14]. ECGs showing an abnormal prolonged QTc were re-checked by an experienced cardiac electrophysiologist. The 1-year follow-up ECG was analysed in the same manner.

### 2.2. Statistical Analysis

Continuous variables were conveyed as a mean ± standard deviation or median with an interquartile range (IQR), and categorical variables as a number and percentage. The Student’s *t*-test and, where applicable, the Mann–Whitney U-test and Wilcoxon signed rank test were performed to compare continuous variables. The chi-squared test was applied to compare categorical variables. A *p*-value of <0.05 was accepted as statistically significant. Univariate and multivariate regression analyses were performed, followed by a multivariate Cox regression analysis (SPSS v27, IBM Corp., Chicago, IL, USA) for variates associated with mortality, and the proportionality of hazard assumption using Cox regression was also tested (SPSS v27, IBM Corp., Chicago, IL, USA).

## 3. Results

**Figure 1:**
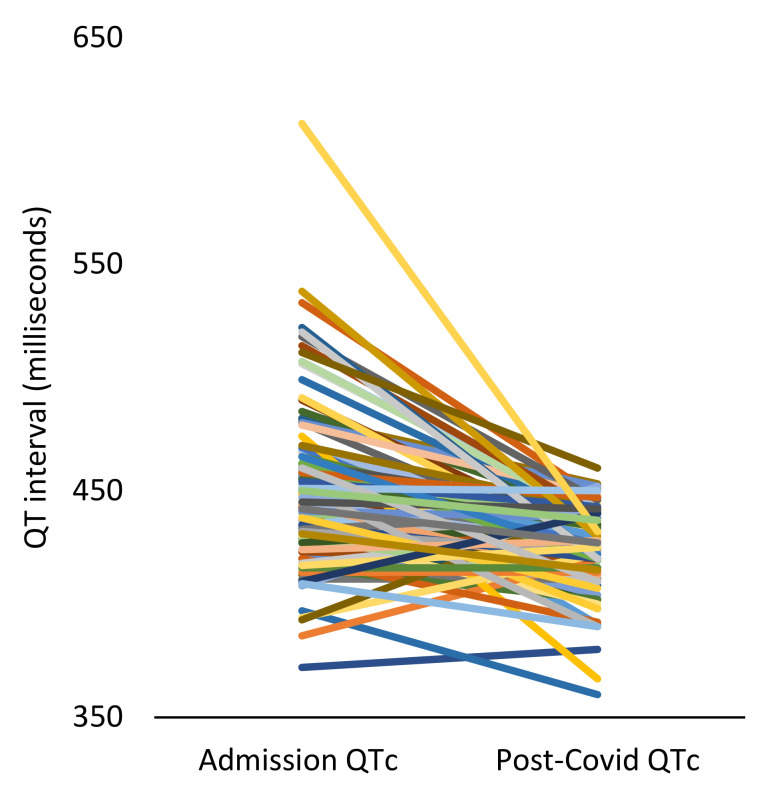
The QTc on admission and in the post-COVID period. The QTc had corrected in the post-COVID period in comparison to the interval recorded on the COVID-19 admission (*p* < 0.01).

Of the 159 patients who had survived the COVID-19 illness during the first wave, 59 (37.1%) had repeat admissions, 57 (35.85%) suffered from long COVID and 28 (17.6%) subsequently died within a year (230.6 ± 154.3 days) of discharge from hospital (Table 1) (*p* < 0.01). Following hospital discharge, 36 (22.6%) patients had documented atrial fibrillation episodes, of which, 25 (69.4%) were a new diagnosis. The baseline QTc (on admission) for the whole cohort was 449.1 ± 33.8 ms, which subsequently shortened to 425.7 ± 18.2 ms within the 1-year follow-up period of COVID illness (Figure 1).

At the 1-year follow-up, patients who had died were older than the patients who remained alive (82.6 vs. 68 years in age, *p* < 0.01), with a similar proportion of male patients in both cohorts (56% vs. 60%, *p* = 0.68). In comparison to the alive group, the deceased had a lower albumin level (37 ± 4.9 vs. 34.4 ± 5.4, *p* = 0.04) and a higher red blood cell distribution width (13.8 ± 1.4 vs. 14.6 ± 1.9, *p* = 0.03) during the COVID-19 admission (Table 2). A similar proportion of the deceased and alive cohorts required an intensive therapy unit (ITU) admission (0% vs. 7.6%, *p* = 0.136), with a similar C-reactive protein level (CRP) (102 vs. 146, *p* = 0.14), and requiring a comparable length of hospital stay (8.4 vs. 8.9 days, *p* = 0.75) with a similar number of repeat hospital admissions (2 [0–2] vs. 0 [0–1], *p* = 0.064) and prevalence of long-COVID (46.2% vs. 42.5%, deceased vs. alive, *p* = 0.8) after the index illness. New onset atrial fibrillation was more common within the deceased cohort (33.3% vs. 12.12%, deceased vs. alive, *p* = 0.006).

From the 159 patients recruited to this study, 103 were identified to have had a repeat ECG within the 1-year follow-up period. A subsequent ECG analysis of the deceased and alive cohorts demonstrated a similar QTc interval at hospitalisation (449.6 ± 27 ms vs. 449.1 ± 34 ms, *p* = 0.93) and following hospital discharge (428.9 ± 18.2 ms vs. 425.7 ± 18.2 ms, *p* = 0.54) for COVID. The QTc did shorten significantly from the index to the subsequent 1-year follow-up interval in the alive (449.1 ms vs. 425.7 ms, *p* < 0.001) and deceased (448.9 ms vs. 428.9 ms, *p* < 0.01) patients. The extent of QTc shortening with recovery from the acute COVID illness was numerically greater in the alive group compared to those who died (−26.01 ± 33.5 ms vs. −20.6 ± 30.04 ms, *p* = 0.5), but did not reach statistical significance (Table 2). The QTc did also significantly shorten between the pre-COVID and the post-COVID intervals in the alive patients (437.01 ms vs. 426.8 ms, *p* < 0.01), although not reaching statistical significance in the deceased group (439 ms vs. 421.5 ms, *p* = 0.06). The degree of QTc correction did not significantly correlate with the albumin level (Pearson’s coefficient = 0.02, *p* = 0.8) or red cell distribution width (RDW) (Pearson’s coefficient = 0.05, *p* = 0.6) during the COVID illness. 

In the overall study group, the R-R interval was 696.4 ± 136.5 on the COVID-19 admission and significantly rose to 811.1 ± 158.9 during the post-COVID period (*p* < 0.01). The R-R interval was statistically similar between the alive and deceased patients on their COVID-19 admission to hospital (691.1 ± 142.3 vs. 717.7 ± 190.5 ms, respectively, *p* = 0.5). In the post-COVID period, the R-R interval was significantly higher in the alive cohort than those who subsequently died (818.9 ± 169.3 vs. 761.1 ± 61.2, *p* = 0.02). 

### Mortality

A univariate logistic regression analysis revealed that age (OR 1.08 [1.04–1.11], *p* < 0.001), RDW during the COVID admission (OR 1.29 [1.09–1.53], *p* = 0.003, albumin level at discharge from hospital for the COVID-19 infection (OR 0.9 [0.83–0.97], *p* = 0.008) and the post-COVID R-R interval (OR 0.995 [0.993–0.998], *p* = 0.002) were predictors for mortality within 1-year of COVID-19 illness. 

**Figure 2:**
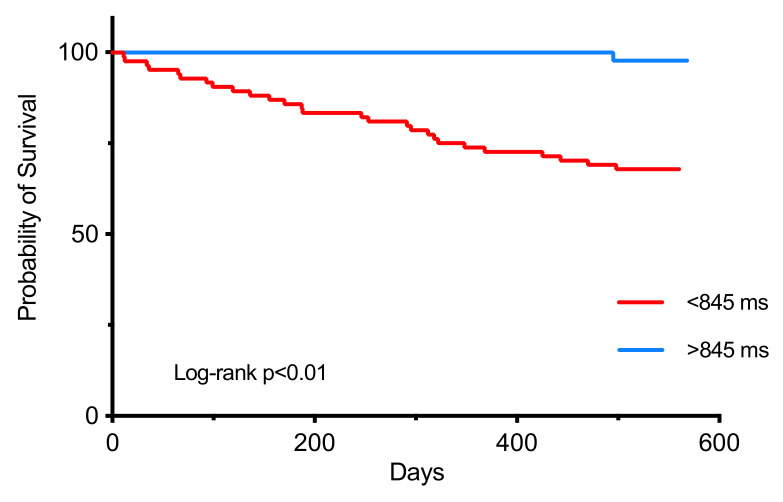
Kaplan–Meier curve comparing patient survival based on the R-R interval. Patients with a resting R-R interval of >845 ms were more likely to die in comparison to patients with an R-R interval of <845 ms (HR 4.5 [2.1–9.6], *p* < 0.01).

A subsequent Cox regression analysis with these variates demonstrated that age (HR 1.098 [1.05–1.15], *p* < 0.01), diabetes (HR 3.972 [1.47–10.8], *p* < 0.01) and the post-COVID R-R interval (HR 0.993 [0.989–0.996], *p* = 0.007) were significantly associated with mortality within 1 year of the COVID illness (Table 3). The ROC analysis revealed an R-R interval of 845 ms as the best cut-off value for predicting survival. Patients with a post-COVID R-R interval of >845 ms were more likely to survive compared to patients with an R-R interval of <845 ms (HR 4.5 [2.1–9.6], *p* < 0.01) (Figure 2).

## 4. Discussion

The QTc is known to prolong with the COVID-19 illness [6,7] and, in this study, we have confirmed that it does shorten with recovery (Figure 1); there is no lasting effect. Interestingly, the QTc shortened in both the alive and deceased groups during the post-COVID period, and reached a shorter interval than the pre-COVID recordings. This is logical, as the association between the QT interval and risk of all-cause mortality is ‘U-shaped’; the mortality risk reduces as the QTc interval shortens towards the mid-quintile, and the highest risk of mortality exists in either extreme (long and short QT) [15]. Therefore, the shortening of the QTc interval to a level shorter than the pre-COVID recording may be indicative of the physiological recovery following the systemic illness. This was, however, not associated with 1-year mortality; the QT-interval is more likely to be a marker of outcome in the acute illness only [7]. 

Our study demonstrates that the post-COVID R-R interval is a predictor of 1-year mortality: a prolonged R-R interval is associated with a low mortality risk (*p* < 0.01). The R-R interval is a surrogate marker for the heart rate (HR) and, therefore, it suggests that the heart rate in the post-COVID period is a predictor of 1-year outcomes; a higher resting HR is associated with mortality. The heart rate (HR) is an established predictor of all-cause mortality in the general population [16], and our study indicates that it can be applied to the COVID-19 illness. This is advantageous, as this basic variable may provide valuable insight toward the overall physiological health following the infection; it is an indicator of recovery. Furthermore, a dichotomous value of 845 ms (HR 71) has been identified as the ideal cut-off value. This may suggest a J-shaped relationship between the heart rate and mortality. This value is also in keeping with previous reports: a HR of >70 was found to be associated with mortality, and reducing it to <70 was associated with improved cardiovascular outcomes, with some benefit in all-cause mortality also [17].

**Figure 3:**
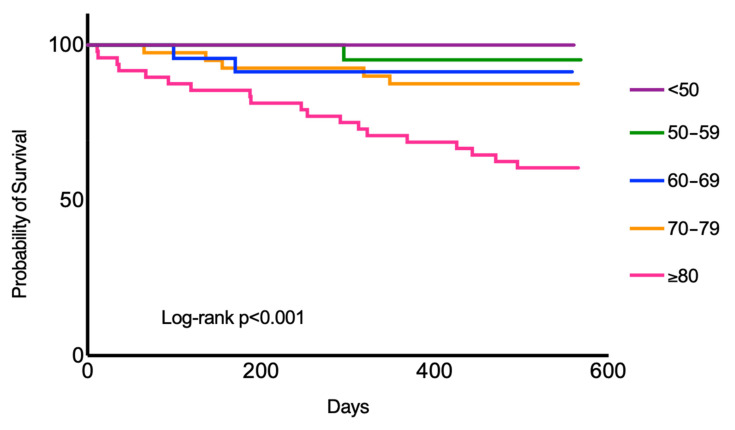
KM survival curve for age groups. The >80-year-old patients were much more likely to perish in the post-COVID period compared to their younger counterparts (HR 3.7 [1.7–8.2], *p* < 0.01).

COVID-19 in the medium-term is associated with ongoing illness [18]; our study corroborates the finding that COVID-19 is also associated with ongoing mortality risk in the medium-term. Within our study population that had survived the COVID illness during the first wave, 17.6% had subsequently perished. This is higher than previous observational studies, which confirm an ongoing mortality risk in the medium post-discharge period, with mortality rates of 12.3% [19] and 13.4% [20]. The higher rate in our study is likely multi-factorial. Other than the significant co-morbidities including age (83 years) in our patients (Figure 3), they perished within 230 days of their index illness. At this stage, the UK was tackling the second COVID-19 wave and therefore it is plausible that the attention was diverted by this emergency from the post-COVID care that these patients required. The deaths may or may not have been a direct consequence of the infection, but the greater prevalence of multi-organ dysfunction and higher proportion of re-admissions in the COVID-19 cohort compared to the general population suggest that there is a link [16]. 

The univariate analysis found that low albumin and a high red cell distribution width could be associated with 1-year mortality. This is a novel finding, as hypoalbuminaemia and a high RDW have been witnessed in unwell COVID-19 patients and are recognised features of poor acute outcomes [21,22]. As the SARS-CoV-2 infection is a systemic disorder, the mechanism behind hypoalbuminaemia and raised RDW is likely similar to other systemic illnesses. Increased inflammation linked to vascular permeability results in albumin leakage to the extravascular space; coupled with malnutrition, it may lead to hypoalbuminaemia [23]. A raised RDW in COVID-19 is hypothesised to occur due to the inflammatory response. There is an up-regulation of pro-inflammatory cytokines in COVID-19 resulting in a reduction in red cell production and delayed clearance [24], which leads to a higher red cell distribution width. It is unlikely that these markers directly contribute to mortality in the medium-term; it is more plausible that they are markers of the disease severity and the overall physiological health in the recovery phase. 

The prevalence of atrial fibrillation in our cohort from the onset of the infection to the end of the 1-year study period was relatively high (22.6%), of which, 69.4% was a new diagnosis. Atrial fibrillation was also significantly higher in the deceased group. The prevalence of new onset AF in patients with the COVID-19 infection has been reported as similarly high in the literature (52.9%) [25]. The number in our series may be associated with the longer follow-up period and represents an ongoing risk of developing AF, even in the post-illness period. This is significant, as AF is associated with poor outcomes in COVID-19 [25]. It also represents a growing population of AF that may require future treatment, including ablation therapy; there is a concern of a wave of patients with new onset AF that will burden healthcare systems across the globe [26]. Management in the acute setting is in accordance with guidelines, including anti-coagulation [27]. The regular monitoring of the QT interval is required, as pharmacotherapy is associated with QTc prolongation, which will be exacerbated by the acute illness [27]. 

The male gender is a recognised risk factor for poor outcomes in hospitalised COVID-19 patients; females may have better outcomes [28,29]. This is thought to be linked to the female sex hormone estradiol (E2) and progesterone, which may have protective roles in COVID-19. Estradiol may suppress the inflammatory chaos characteristic of the SARS-CoV-2 infection whilst enhancing the anti-viral immune response. It also upregulates the angiotensin converting enzyme-2 (ACE2) expression and therefore counteracts the effects of the SARS-CoV-2 virus, which naturally promotes the down-regulation of this enzyme [28], an effect contributing to acute respiratory failure. Progesterone may also have a significant pulmonary protective function; in mouse models, it was found to soften the inflammatory response and promote healing in the lungs. Our study did not find an association between gender and unfavorable outcomes. This is likely to be multi-factorial. We evaluated the medium-term outcomes in our cohort, and it may be that the protective effects seen in female patients may only apply to the acute viral illness, and therefore during acute hospitalisation; it may not have the same benefit in the medium-term. In addition, our patient cohort was relatively elderly, and therefore the majority of the female patients in this study were post-menopause; consequently, the hormonal protective benefits seen in females may well have been negated. 

### Limitations

The findings of this study cannot be applied to very young patients with COVID-19, as the average age of this study cohort is advanced (70 years of age). Confounding variables potentially affecting the ECG parameters cannot be excluded due to the retrospective nature of this study. For instance, the interval at which the ECGs were taken in the post-COVID period varied amongst the patients, which may have implications for the ECG measurements; the QTc and R-R intervals are dynamic and can be affected by a variety of factors. The corrected QT interval was measured using Bazett’s formula, which can over-estimate the QTc at high heart rates; however, it is a consistent approach and should not affect the outcomes related to this study.

## 5. Conclusions

The mortality risk with the COVID-19 illness extends to the post-COVID recovery period. The QTc interval does recover in the post-COVID phase, whilst the R-R interval is a valuable indicator of 1-year mortality. 

## Figures and Tables

**Table 1:** Overall demographics of patients who had survived the index COVID-19 illness, including post-COVID data.

COVID Admission	
N=	159
Age (years)	70.5 ± 16.5
Male	94 (59.1%)
ITU admission (patients)	10 (6.3%)
Hospital stay (days)	8.85 ± 7.9
Diabetes	44 (27.7%)
Hypertension	94 (59.1%)
Ischaemic heart disease	17 (10.7%)
Cancer	21 (13.2%)
Dementia	17 (10.7%)
Chronic kidney disease	23 (14.5%)
Left ventricle ejection fraction (%)	55.5 ± 6.5
Haemoglobin (g/L)	117.7 ± 20.7
Red cell distribution width (%)	13.9 ± 1.5
Albumin (g/L)	36.6 ± 5.1
C-reactive protein (mg/L)	143.2 ± 101
Troponin (ng/L)	413.4 ± 2815
Pre-COVID admission QTc (ms)	435.25 ± 25.6
QTc on COVID admission (ms)	449.1 ± 33.8
R-R interval on COVID admission (ms)	696.4 + 136.5
Post-COVID:	
Long COVID	57 (35.8%)
Repeat admissions (patients)	59 (37.1%)
New atrial fibrillation in the 1-year follow-up	25 (15.7%)
1-year mortality	28 (17.6%)
Follow-up to death from admission (days)	230.6 ± 154.3
Post-COVID QTc (ms)	425.7 ± 18.2
R-R interval post-COVID (ms)	811.1 ± 158.9

**Table 2:** A comparison of patients who, at 1-year follow-up, were either alive or deceased. The deceased cohort was older, with a shorter post-COVID R-R interval.

	Alive	Deceased	*p*-Value
N=	131	28	
Age (years)	68 ± 16	83 ± 10.7	<0.001
Female gender	52 (39.6)	13 (46.4)	0.679
COVID ITU admission	10 (7.6)	0 (0)	0.136
COVID hospital stay (days)	8.9 ± 7.83	8.4 ± 8.4	0.75
Number of repeat admissions	0 (0–1)	2 (0–2)	0.064
Long COVID (*n* = 133)	51 (42.5)	6 (46.2)	0.8
New atrial fibrillation	16 (12.2)	9 (32.1)	0.006
Diabetes	33 (25.2)	11 (39.2)	0.096
Hypertension	78 (59.5)	16 (57.1)	0.68
Ischaemic heart disease	15 (11.5)	2 (7.1)	0.544
Cancer	18 (13.7)	3 (10.7)	0.724
Dementia	12 (9.2)	5 (17.9)	0.45
Chronic kidney disease	16 (12.2)	7 (25)	0.21
Left ventricle ejection fraction (%)	55.9	52.3	0.33
Lab values:			
Troponin (ng/L)	581.3 ± 3359.3	51.3 ± 79.6	0.014
Haemoglobin (g/L)	118.1 ± 20.1	116.4 ± 23.2	0.7
Red cell distribution width (%)	13.8 ± 1.4	14.6 ± 1.8	0.012
Albumin (g/L)	37.1 ± 4.9	34.1 ± 5.4	0.02
C-reactive protein (mg/L)	146.9 ± 102.5	121.9 ± 94.6	0.2
ECG:			
QTc pre-admission (ms)	434.4 ± 25.4	439 ± 27.6	0.48
QTc on admission (ms)	449.1 ± 34	449.6 ± 27	0.93
QTc post-COVID (ms)	425.7 ±18.2	428.9 ± 18.5	0.54
Post-COVID QRS duration (ms)	96.5 ± 18.9	94.8 ± 19.8	0.75
R-R interval on COVID admission (ms)	691.1 ± 142.3	717.7 ± 190.5	0.5
Post-COVID R-R interval (ms)	818.9 ± 169.3	761.1 ± 61.2	0.02
QTc change during follow-up (ms)	−26.01 ± 33.5	−20.6 ± 30.04	0.5

Values are mean ± standard deviation, *n* (%), or median (interquartile range).

**Table 3:** Variates associated with mortality.

	Odds Ratio	95% Confidence Interval	*p*-Value
Univariate Analysis			
Age	1.075	1.04–1.113	<0.001
Diabetes	1.78	0.832–3.8	0.137
Chronic kidney disease	2.09	0.89–4.92	0.091
Red cell distribution width	1.29	1.091–1.525	0.003
Albumin on discharge	0.897	0.827–0.973	0.008
C-reactive protein	0.997	0.994–1.001	0.2
Post-COVID QTc	1.02	0.991–1.05	0.19
Post-COVID R-R interval	0.995	0.993–0.998	0.002
Multivariate Cox regression analysis			
	Hazard Ratio	95% Confidence Interval	*p*-Value
Age	1.098	1.045–1.153	<0.01
Diabetes	3.972	1.47–10.8	<0.01
Post-COVID R-R interval	0.993	0.989–0.996	0.007

## Data Availability

Data is on file and available upon reasonable request.
